# The Commonly Utilized Natural Products during the COVID-19 Pandemic in Saudi Arabia: A Cross-Sectional Online Survey

**DOI:** 10.3390/ijerph18094688

**Published:** 2021-04-28

**Authors:** Sina M. AlNajrany, Yousif Asiri, Ibrahim Sales, Yazed AlRuthia

**Affiliations:** 1 Chronic Disease Prevention Centre, Saudi Ministry of Health, Riyadh 11451, Saudi Arabia; Salnajrany@moh.gov.sa; 2 Department of Clinical Pharmacy, College of Pharmacy, King Saud University, Riyadh 11451, Saudi Arabia; yasiri@ksu.edu.sa (Y.A.); isales@ksu.edu.sa (I.S.); 3 Pharmacoeconomics Research Unit, College of Pharmacy, King Saud University, Riyadh 11451, Saudi Arabia

**Keywords:** herbal medicine, Saudi Arabia, COVID-19, prescription drugs, dietary supplements

## Abstract

Objectives: The utilization rate of herbal and dietary supplements among the Saudi population is reported to be high. However, the utilization rate and types of herbal and dietary supplements during the COVID-19 pandemic are largely unknown. Methods: This was an online questionnaire-based cross-sectional study that used convenience sampling and social media platforms (Telegram^®^, Twitter^®^, and WhatsApp^®^) to disseminate a 12-item questionnaire across the Saudi general public aged 18 years and above. The questionnaire inquired about the sociodemographic characteristics (e.g., age, gender, education, geographical location), presence of chronic health conditions and the use of prescription medications, awareness of the viral nature of COVID-19 infection and its common symptoms, and the commonly utilized herbal and/or dietary supplements. Results: Sixty-four percent of the 1473 participants reported using herbal and/or dietary supplements for the purpose of boosting their immune system to prevent COVID-19 infection. In addition, 88.2% of the respondents were misinformed about the manifestation of COVID-19 symptoms. Most of the participants were Saudi (91.9%), aged 49 years and younger (83%), female (52%), and healthy (81%). Honey (46%), lemon (45%), ginger (36%), vitamin C (32%), black seed (26%), garlic (26%), and turmeric (19%) were the most commonly used herbal and/or dietary supplements by the participants. Saudi nationals (OR = 1.67, 95% CI: 1.08 to 2.6, *p* = 0.02), older adults (OR = 1.30, 95% CI:1.10 to 1.55, *p* = 0.002), and those taking prescription medications had higher odds of using dietary and/or herbal supplements (OR = 3.31, 95% CI: 2.61 to 4.18, *p* < 0.0001). Conclusion: The utilization rate of herbal and dietary supplements among the Saudi public during the COVID-19 pandemic is high. Future studies should examine the impact of different public awareness campaigns aimed at improving the public knowledge of the risk and benefits associated with the use of different commonly utilized herbal and dietary products identified in this study.

## 1. Introduction

In December 2019, pneumonia associated with the coronavirus disease 2019 (COVID-19) emerged in Wuhan, Hubei Province, China, and has spread rapidly ever since to the rest of the world [1,2,3,4,5,6]. In Saudi Arabia, the first COVID-19 case was registered on 2 March 2020 [2], and the World Health Organization (WHO) declared COVID-19 a pandemic on 11 March 2020 [3,4]. As of 27 February 2021, the cumulative number of confirmed COVID-19 cases in Saudi Arabia was 377,000 with 368,000 recovered cases and 6483 deaths [7,8]. In the absence of effective treatments and vaccines for COVID-19, the public health sectors in almost all countries have taken different preventive measures to contain the infection, such as quarantine measures, social distancing, and face masks [9].

One of the most commonly utilized measures to boost immunity and protect against COVID-19 infection in the Far, Near, and the Middle East is the use of herbal and complementary medicine [10,11]. According to the National Center for Complementary and Integrative Health, natural products (NP) are generally classified as dietary supplements and include products such as herbs, vitamins and minerals, and probiotics [12]. Although there is not strong scientific evidence that supports any claim of efficacy of herbal supplements in the management of viral infections such as COVID-19, their use is prevalent in many countries, especially in the Far East [10,11,12,13,14]. These practices continue despite the fact that the use of herbs and bioactives has the potential to lead to pharmacokinetic or pharmacodynamic changes in many commonly used medications, particularly in the elderly [15]. Moreover, the WHO reported that the use of traditional and complementary medicine varies widely throughout the world and ranges from 1% to 99% of the population depending upon the country and local customs [16]. In Saudi Arabia, 75.6% of the general public in Riyadh reported using herbal medicines [17]. This high utilization rate of herbal medicines among the general public was attributable to the common belief that herbal medicines are generally safe [17,18,19]. Additionally, it has been reported in a study, which surveyed a sample of herbal medicine consumers in Saudi Arabia, that herbal medicines are generally harmless and safe [18]. Black cumin (40.4%), honey (58.6%), and dietary supplements (e.g., vitamins and minerals) (59.4%) were the most commonly used supplements according to a study the explored the use of complementary and alternative medicine (CAM) among a sample of 736 adolescents in the city of Al Khobar, Saudi Arabia. Medicinal herbs, such as myrrh and ginseng, were reported to be used by 37% of the surveyed adolescents, and females reported a higher rate of utilization and more favorable views of medicinal herbs in comparison to their male counterparts [20]. Furthermore, ginger and cinnamon were utilized most frequently among females according to a community-based survey in the central region in Saudi Arabia [19]. Additionally, participants reported that they used CAM for joint pain and chronic disease states such as diabetes, hypertension, and dyslipidemia [19].

Global use of herbal medicines and dietary supplements during the COVID-19 pandemic has increased significantly and has been used both in combination with prescription medications and alone [21,22,23,24,25]. According to a meta-analysis that included seven randomized controlled trials that evaluated the efficacy of herbal medicines in the resolution of COVID-19 symptoms, such as fever and dry cough, the combination of herbal medicines and prescription drugs resulted in shorter time to recovery from these symptoms [21]. In China, the use of herbal medicines is claimed to reduce the rate of infection according to a review article that presented anecdotal evidence from various Chinese medicine programs in 23 Chinese provinces [22]. In Saudi Arabia, the rate of herbal medicine utilization during the COVID-19 pandemic was reported to be 22.1% according to a cross-sectional questionnaire-based study that explored the public knowledge of the preventive measures of COVID-19 and their beliefs about the use of herbal medicines for COVID-19 prevention. However, the study did not explore the commonly utilized herbal products among the Saudi population for the COVID-19 prevention [26]. Therefore, this study aimed to explore the most commonly utilized NP during the COVID-19 pandemic and the predictors of utilizing NP, such as the sociodemographic characteristics and knowledge about the COVID-19 infection among the general public in Saudi Arabia.

## 2. Methods

### 2.1. Study Design and Data Collection

This was an online questionnaire-based cross-sectional study that was conducted between 29 March and 31 August 2020. The questionnaire consisted of 12 questions that inquired about the characteristics of the participants (e.g., age, gender, employment status, regional location within Saudi Arabia, educational level, nationality, presence of chronic health conditions, and the use of prescription medications) as well as the most commonly utilized NP in Saudi Arabia. Participants were also asked about their awareness of the viral nature of COVID-19 infection and its most common symptoms (e.g., fever, cough, shortness of breath, vomiting, and diarrhea). The questionnaire was developed based on an extensive literature review of the most commonly utilized NP in Saudi Arabia [21,22,23], and reviewed by the first and last authors to check its content and face validity (Appendix A). Furthermore, open-ended questions were included to allow the participants to write any NP not mentioned in the questionnaire. Additionally, the questionnaire was pilot tested among a group of 30 people from the general public to check its comprehensibility and usability. Those aged ≥18 years and living in Saudi Arabia were included in the study. Participants who did not complete the questionnaire were excluded as well as those under 18 years of age. Convenience sampling was used, and eligible individuals were sent an invitation to participate through different social media platforms (Telegram^®^, Twitter^®^, and WhatsApp^®^). The questionnaire was administered in Arabic since the majority of the population in Saudi Arabia are native Arabic speakers.

### 2.2. Statistical Analysis

The characteristics of the participants (e.g., age, nationality, geographic location, educational level, employment status, and presence of chronic health conditions) were presented using descriptive statistics, such as frequencies and percentages. Logistic regression analysis was conducted to examine the association between the use of NP and different participants’ characteristics, such as nationality, gender, education, employment status, presence of chronic health conditions, awareness of the viral nature and common symptoms of COVID-19, and the utilization of prescription medications. The minimum sample size was estimated to be 1302 individuals for multiple logistic regression with up to nine predictors, α = 0.05, β = 0.2, power of 0.95, and a small effect size of Cohen’s F^2^ = 0.01. All the statistical analyses were conducted using SAS software, version 9.4 (SAS Institute Inc, Cary, NC, USA).

## 3. Results

**Figure 1:**
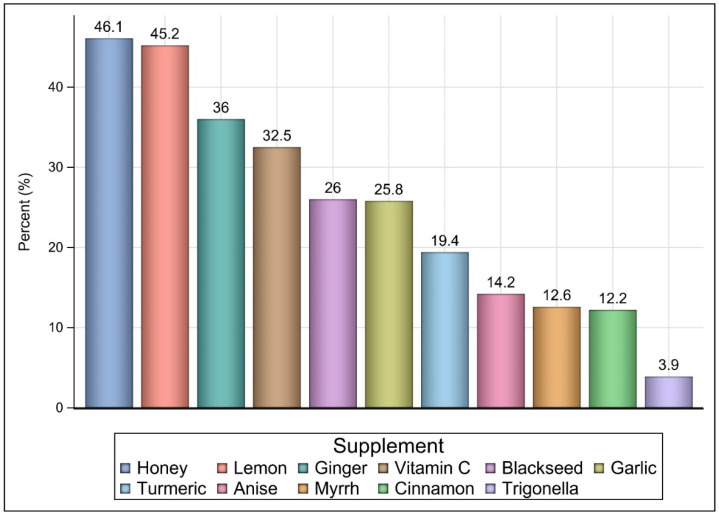
The most commonly utilized supplements among NP users (n = 955).

**Figure 2:**
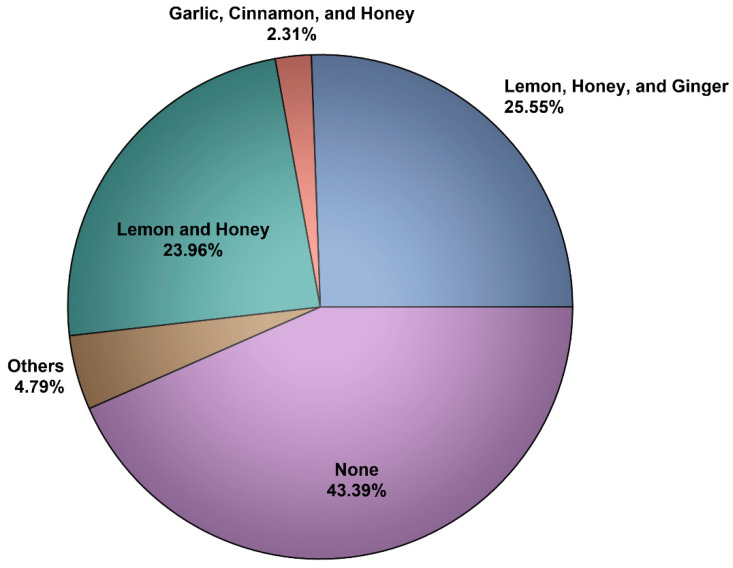
The most commonly utilized NP mixtures among NP users (n = 955).

The number of participants who filled the online questionnaire was 1488. However, 1473 participants (98.99%) met the inclusion criteria (e.g., completed the questionnaire and aged ≥18 years) and were included in the analysis. Most of the participants were female (58.9%), Saudi (91.9%), ≥30 years (68.97%), had a Bachelor of Science degree or higher (77.66%), employed (62.38%), from the central region (53.3%), and had no chronic health conditions (81%) as shown in Table 1. The majority of the participants (86.7%) believed that the COVID-19 infection symptoms are only fever and respiratory (e.g., cough, shortness of breath, and fever), 1.43% (n = 21) believed the symptoms are only fever and gastrointestinal (e.g., diarrhea, nausea, and vomiting), and 11.8% (n = 175) correctly believed that the symptoms can be fever, respiratory, gastrointestinal, and nonspecific (e.g., headache, loss of taste or smell). Most of the participants (98.57%) correctly believed that the COVID-19 infection is viral, while only 1.43% believed that the infection is bacterial. Sixty-four percent (n = 955) of the participants reported using NP during the COVID-19 pandemic because they think it might boost their immunity against the infection. Honey (46.1%), lemon (45.2%), ginger (36%), vitamin C (32.5%), black seed (26%), and garlic (25.8%) were the most commonly utilized NP among NP users (n = 955) as shown in Figure 1. Besides the commonly utilized NP that were included in the questionnaire, some participants (2.5%) reported using other NP, such as mint, onions, *Saussurea costus*, thyme, clove solution, chamomile, cumin, moringa, and fennel. Lemon with honey and ginger as well as lemon with honey were the most commonly utilized NP mixtures among NP users (n = 955) as shown in Figure 2. A few participants (4.79%) reported using other NP mixtures, such as honey with black seed, lemon with honey and ginger, turmeric with garlic and hot pepper, and ginger with mint and saffron. The percentage of participants who reported using more than one NP during the pandemic was 57.03% from the total number of participants, and 87.96% from those who reported utilizing NP during the COVID-19 pandemic.

Table 2 shows that the association between the use of NP and different factors, such as age, nationality, education, employment status, presence of comorbidities, awareness of the viral nature of the COVID-19 infection, awareness of the commonly encountered COVID-19 infection symptoms, and taking prescription medications in a multivariable regression analysis. Saudi nationality was associated with higher odds of using NP (OR = 1.67, 95% CI: 1.08 to 2.6, *p* = 0.02). Every 10-year increase in age was associated with 30.3% higher odds of using NP (OR= 1.30, 95% CI:1.10 to 1.55, *p* = 0.002). For example, the percentage of NP users among those aged 18-29 years was 55.36% in comparison to 68.32% among those aged 30-49 years (*p* < 0.0001). Those taking prescription medications had higher odds of using NP in comparison to their counterparts who are not taking prescription medications (OR= 3.31, 95% CI: 2.61 to 4.18, *p* < 0.0001). 

## 4. Discussion

The utilization of NP for various reasons is prevalent in Saudi Arabia. According to a previously published study that explored the extent of herbal medicine utilization among a randomly selected sample of 809 adults from the general public in Saudi Arabia, 88.4% of the surveyed people reported using herbal medicines at some point in time, and the majority of them (88.7%) reported using these supplements for therapeutic reasons [27]. This high rate of NP utilization among the general public exceeded the one found in this study during the COVID-19 pandemic, which was 64%. Although the study did not explore the reasons behind the use of NP, it is largely believed, based on the findings of previously published studies that explored the use of complementary and alternative medicines during pandemic times, that the reason behind the use of these supplements is mostly preventative [25,26,28,29].

Different NP are used for prophylactic and treatment reasons against viral and bacterial infections [29]. For example, the herbal products used for prophylaxis against COVID-19 in Morocco are more diverse than the ones used in Saudi Arabia [25]. *Allium Sativum*, *Olea europaea*, *Allium cepa*, *Zingiber officinale*, *Thymus maroccanus*, and *Eucalyptus globules* are some examples of the commonly utilized herbal products by Moroccan families during the COVID-19 pandemic according to a recently published questionnaire-based cross-sectional study [24]. In Saudi Arabia, *Trigonella foenum-graecum*, *Pimpinella anisum*, *Nigella sativa*, green tea, and *Peganum* were found to be commonly utilized among patients with chronic health conditions in the city of Bisha. Moreover, honey, black-seed, myrrh, mint, ginger, and green tea were found to be commonly utilized among the Saudi population for nutritional and therapeutic purposes in multiple studies conducted in different Saudi cities [30,31,32,33,34]. As reported in our study, the public has focused on specific NP for the prevention of COVID-19 and placed greater emphasis upon using some types with the exclusion of others. This may be due to the proliferation of scientific publications that have implicated a benefit of using particular types of NP in the prevention and/or treatment of COVID-19 [35,36,37,38,39]. However, no study has so far explored the NP that are commonly used during the COVID-19 pandemic for prophylactic purposes. Alyami et al. has explored the public knowledge of preventive measures against COVID-19 as well as knowledge of the COVID-19 infection and the rate of herbal medicine utilization among the general public in Saudi Arabia [26]. The reported rate of herbal medicine utilization during the pandemic was 22.1% which is much lower than the one found in this study. The large difference in NP usage is surprising due to the similar participant demographics between the two studies. A possible reason could be due to the fact that our study was conducted for a longer period of time. The study by Alyami et al. lasted until 6 June 2020, which was still within the lockdown period [26]. Our study lasted over a month beyond the end of the lockdown period. Hence, the participants in our study had more access to NP and may have been more inclined to seek alternative methods of prevention due to the unexpected long duration of the pandemic. Moreover, the respondents’ knowledge of COVID-19 infection was found to be moderate in comparison to the 98% of the surveyed participants in this study who believed that the COVID-19 infection is viral. Additionally, at least 20% of the participants in the Alyami et al. study reported that ginger, vitamin C, onions, and garlic have prophylactic activity against COVID-19 infection [26]. Interestingly, we found that ginger, vitamin C, and garlic were utilized by at least 25% of the surveyed participants. Furthermore, our study explored for the first time the different NP that are commonly utilized by the public in Saudi Arabia during the COVID-19 pandemic. The Saudi nationality was found to be associated with higher odds of using NP. This confirms the high rate of NP utilization among the Saudi population which has been previously reported [30,31,32,33,34]. Furthermore, older adults and those taking prescription medications had higher odds of using NP in comparison to their young and healthy counterparts. This high rate of NP, especially among older adults and those taking prescription medications, may increase the risk of serious herb–drug interactions and jeopardize the therapeutic efficacy of many medications used for the treatment of various chronic health conditions [40]. Furthermore, these risks may offset the potential benefit of shortening the course of COVID-19 infections [21]. Therefore, similar to the lessons learned from the Middle East Respiratory Syndrome (MERS) and early COVID-19 responses in Saudi Arabia, multiple educational campaigns should be launched to educate the public about the potential herb–drug interactions [40]. In addition, focus should be placed upon emphasizing the need of individuals to control their health conditions during this unconventional pandemic time given the high prevalence of chronic health conditions among the Saudi population.

Although this is one of the few studies that explored the utilization rate and different commonly used NP among the Saudi population, the study has several limitations that must be acknowledged. First, this was a cross-sectional questionnaire-based study which is subject to information bias [41]. Secondly, selection bias cannot be excluded given the fact that most participants were under the age of 50 years, and those aged 50 years and above who were found to have higher odds of using NP represented only 17% of the study sample. Additionally, nonresponse bias cannot be excluded due to the fact that the questionnaire was disseminated across different social media platforms [42]. Moreover, this was not a nationally representative study; therefore, the generalizability of the findings is questionable due to the convenience sampling that was used in this study. Furthermore, the study did not examine the association between health literacy and the utilization rate of NP. Finally, no distinction was made between those using NP previously or currently for curative purposes and participants utilizing them exclusively for preventative reasons.

## 5. Conclusions

The utilization rate of NP during the COVID-19 pandemic is high among the Saudi public. Different NP with high potential of drug–herb interactions have been reported to be used by a significant percentage of the surveyed participants. Public awareness campaigns should be launched to educate the public about the benefits and risks of using different NP, especially those identified in this study.

## Figures and Tables

**Table 1:** Demographic characteristics of the participants (n = 1473).

Characteristics	n (%)
Gender	
Male	605 (41.1)
Female	868 (58.9)
Age categories (year)	
18–29	456 (31.0)
30–49	767 (52.1)
50–64	220 (14.9)
≥65	29 (2.0)
Nationality	
Saudi	1353 (91.9)
Non-Saudi	120 (8.1)
Geographic location	
Central region	785 (53.3)
Eastern region	136 (9.2)
Western region	473 (32.1)
Northern region	26(1.8)
Southern region	53(3.6)
Education level	
Intermediate school diploma	36 (2.4)
High school diploma	175 (11.9)
Associate degree	118 (8.0)
Bachelor of Science degree	764 (51.9)
Postgraduate degrees (e.g., Master of Science or Doctor of Philosophy)	380 (25.8)
Employment status	
Employed	919 (62.4)
Unemployed	554 (37.6)
Chronic health conditions	
Yes	280 (19.0)
No	1193(81.0)
Taking Prescription medications	
Yes	310 (21.05)
No	1163 (78.95)

Data are presented as N (%).

**Table 2:** Multiple logistic regression analysis for the use of NP.

Variable	Odds Ratio (OR)	95% Confidence Interval (CI)		*p*-Value
		Lower CI	Upper CI	
Nationality (Saudi)	1.678	1.082	2.6	0.020 *
Female gender	0.916	0.718	1.168	0.477
Age	1.303	1.1	1.545	0.002 *
Education	1.038	0.936	1.15	0.479
Employment status (Unemployed)	0.922	0.722	1.176	0.512
Presence of comorbidities	0.924	0.682	1.254	0.613
Awareness of the viral nature of COVID-19 infection	3.345	0.945	11.846	0.061
Awareness of the common COVID-19 symptoms	1.114	0.786	1.58	0.542
Taking prescription medication	3.31	2.61	4.18	<0.0001 *

* *p* < 0.05.

## Data Availability

Study data are available from the corresponding author on reasonable request.

## Appendix A

(1) **Nationality:**
  ○ Saudi
  ○ Non-Saudi
(2) **Residence area:**
  ○ Central region
  ○ Eastern region
  ○ Western region
  ○ Northern region
  ○ Southern region
(3) **Gender:**
  ○ Male
  ○ Female
(4) **Age**:
  ○ 18–29 years old
  ○ 30–49 years old
  ○ 50–64 years old
  ○ 65 years old and above
(5) **Educational level:**
  ○ Intermediate or middle school diploma
  ○ Secondary or high school diploma
  ○ Associate degree (e.g., community college).
  ○ Bachelor’s degree
  ○ Post-graduate education (e.g., master or doctorate of philosophy degrees (PhD))
(6) **Employment status:**
  ○ Employed
  ○ Unemployed
(7) **Health status:**
  ○ Healthy (e.g., no chronic health conditions such as diabetes and hypertension).
  ○ Have chronic health conditions (e.g., diabetes, hypertension, dyslipidemia, asthma and others).
(8) **What type of infection is COVID-19?**
  ○ Viral infection
  ○ Bacterial infection
(9) **What are the main symptoms of this infection?**
  ○ Respiratory symptoms (fever, cough, shortness of breath).
  ○ Gastrointestinal symptoms (fever, diarrhea, and vomiting).
  ○ Fever, respiratory, gastrointestinal, and nonspecific (e.g., loss of taste and/or smell, fatigue, headache) symptoms.
(10) **Do you use any of the following herbs to protect yourself against COVID-19 infection? (You can pick more than one).**
  ○ Ginger
  ○ Cinnamon
  ○ Anise
  ○ Honey
  ○ Garlic
  ○ Lemon
  ○ Black seeds
  ○ Myrrh
  ○ Turmeric
  ○ Trigonella
  ○ I don’t use any herbs
  ○ Others (specify it).
(11) **Do you use any of the following herbal mixtures? (You can pick more than one).**
  ○ Lemon with Honey and Ginger.
  ○ Garlic with Cinnamon and Honey.
  ○ Lemon with Honey.
  ○ I don’t use any herbal mixtures.
  ○ Others (specify it).
(12) **Do you use any prescription medications?**
  ○ Yes
  ○ No
